# Reestablishment of Occlusal Vertical Dimension in Complete Denture Wearing in Two Stages

**DOI:** 10.1155/2015/762914

**Published:** 2015-10-26

**Authors:** Danny Omar Mendoza Marin, Andressa Rosa Perin Leite, Norberto Martins de Oliveira Junior, Marco Antonio Compagnoni, Ana Carolina Pero, João Neudenir Arioli Filho

**Affiliations:** Department of Dental Materials and Prosthodontics, Araraquara Dental School, UNESP, Paulista State University, Humaitá Street 1680, 14801-903 Araraquara, SP, Brazil

## Abstract

The assessment and reestablishment of the occlusal vertical dimension (OVD) are considered important factors in the treatment of complete denture wearers. The long-time use of a complete denture can result in jaw displacement due to abrasion of the artificial teeth and residual ridge resorption, causing esthetic complications. Most patients with old dentures and incorrect OVD accept reestablishment of the OVD with new complete dentures, even if they were used to their old dentures. The present clinical report describes a method of gradual reestablishment of OVD using a diagnostic acrylic splint on artificial teeth in old complete dentures before the manufacture of new complete dentures. *Clinical Significance*. The use of a reversible treatment for reestablishment of the OVD in old complete dentures with a diagnostic occlusal acrylic splint allows for the reestablishment of the intermaxillary relationship, providing physiological conditions of masticatory performance associated with the recovery of facial esthetics in edentulous patients.

## 1. Introduction

The establishment of appropriate occlusal vertical dimension (OVD) is a fundamental factor in the manufacture of new complete dentures in harmony with the patient's masticatory system [1, 2]. However, the long-time use of the same complete denture can result in jaw displacement due to abrasion of the artificial teeth and residual ridge resorption, causing a decrease of the OVD [1, 3–6].

The decrease of the OVD can compromise the esthetic and cause morphologic changes in the complete denture wearers, such as hyperactivity or hypoactivity of the masticatory muscles, increase or decrease in masticatory force, temporomandibular disorders, decreased facial height as a result of mandibular ridge resorption and a downward and forward rotation of the mandible [1, 5, 6], and increasing mandibular prognathism [6]. These complications can significantly affect the function and comfort, as well as the phonetics and esthetic of the patient [3, 7, 8].

The reestablishment of the OVD is considered one of the most challenging and complex procedures during oral rehabilitation of edentulous patients [9]. It is related to the head and cervical spine postures and anterior facial height [10], the esthetics of the entire face and mandibular position, and reestablishment of the masticatory function and phonetics [11, 12].

For this reason, it is usually believed that changes in the OVD should be conservative and for a trial period, with an interim prosthesis if necessary [13], especially before the manufacture of a new complete denture set. One technique that has advocated for reestablishment of OVD includes the use of an acrylic splint in an appropriated OVD [10]. This process allows assessing the patient's tolerance, esthetics, and phonetics at the proposed restored OVD before irreversible changes in the natural dentition or with a new partial denture [13]. This technique has also been described for the edentulous patient with old complete dentures [14].

The present case report describes a method of gradual reestablishment of an appropriate OVD using a diagnostic acrylic splint on artificial teeth in an old complete denture before manufacture of a new complete denture.

## 2. Case Description

A 65-year-old woman came to the Araraquara Dental School, UNESP, Paulista State University, Araraquara, São Paulo, Brazil, for assessment and manufacture of a new complete denture. The complete dentures were fabricated 23 years ago and her principal complaint was poor esthetics and ear pain. Intraoral and extraoral examinations were performed that included supporting structures, oral hygiene, temporomandibular joints, and facial height and symmetry with the OVD, the physiologic rest position, esthetics, and phonetics.

The initial examination revealed that the patient had been wearing a maxillary and a mandibular denture with excessive wear of the artificial teeth, causing decreased OVD and, consequently, an excessive freeway space, downward turn of the commissures, and overrotation and protrusion of the mandible with the dentures in position. In addition, the patient had been experiencing moderate pain in the temporomandibular joint.

An occlusal vertical dimension assessment was performed, according to customary procedures at the department [15], as follows: the patient was instructed to reach a resting position after licking the lips, swallowing, and saying /m/ without occlusion of the artificial teeth. This procedure was repeated 3 times. Mean distance from chin to base of the nose, minus 3 mm, was considered the OVD. Measurements were made using calipers. Following these measurements, an esthetic appraisal was done with occlusion of the artificial teeth in position. Finally, OVD was assessed using a phonetic method [16]. The occlusion of the artificial teeth was not allowed to touch each other during pronunciation of the sibilant sounds.

After determining the OVD through the association of the metric, phonetic, and esthetic methods, the need of 14 mm for the reestablishment of the OVD was demonstrated in the current prostheses. Due to the clinical conditions and the needs of the patient, an oral rehabilitation was proposed in two stages, commencing with using occlusal acrylic splints on the old dentures, like a pretreatment of the OVD, before the manufacture of the new complete denture set.

**Figure 1:**
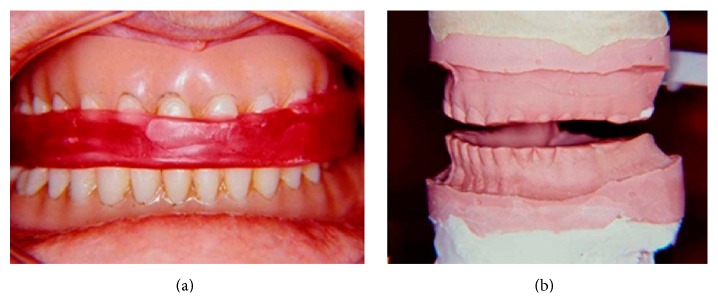
Record for reestablishment of 7 mm in the OVD. (a) Record in wax with 7 mm; (b) first master cast set mounted in a semiadjustable articulator.

**Figure 2:**
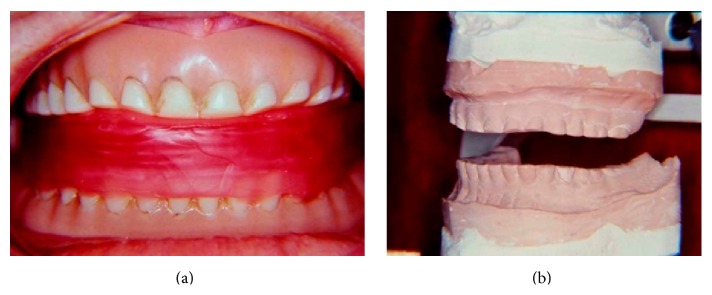
Record for reestablishment of 14 mm in the OVD. (a) Record in wax with 14 mm; (b) second master cast set mounted in a semiadjustable articulator.

Three impressions with alginate (Hydrogum, Zhermack, Badia Polesine, Italy) of the complete denture (1 maxillary denture and two mandibular dentures) were performed and poured with special plaster type IV (Vel-Mix Stone, Kerr Corporation, Orange, California, USA). Next, two occlusal records with a wax roller were taken (one reestablishing the OVD with 7 mm and the other with 14 mm) for reestablishment of the OVD in two stages. All master casts were mounted in a semiadjustable articulator (Bio-Art Equipamentos Odontológicos Ltda.©, São Carlos, São Paulo, Brazil) for the manufacture of the occlusal acrylic splints. First, the maxillary master cast was mounted in the semiadjustable articulator and, after that, each mandibular master cast, with the respective record (7 mm and 14 mm), was mounted using the same maxillary master cast (Figures 1 and 2).

**Figure 3:**
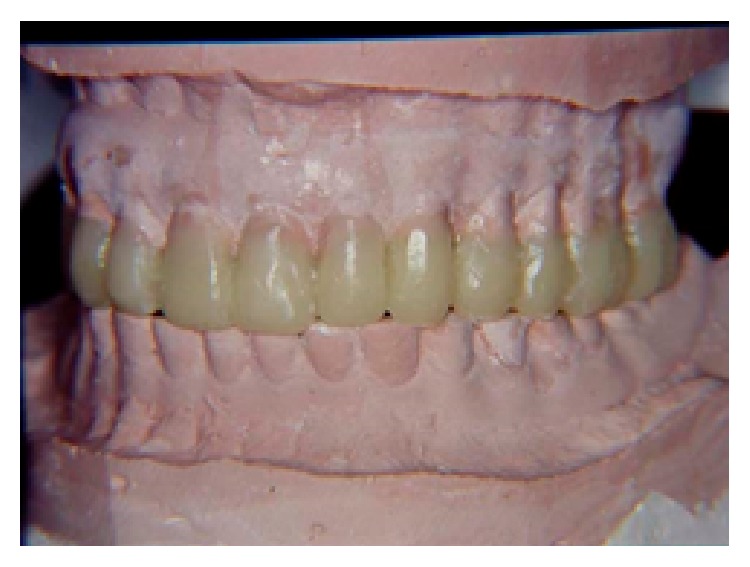
Waxing of the maxillary cast for manufacturing a maxillary occlusal splint for a 7 mm increase in OVD.

**Figure 4:**
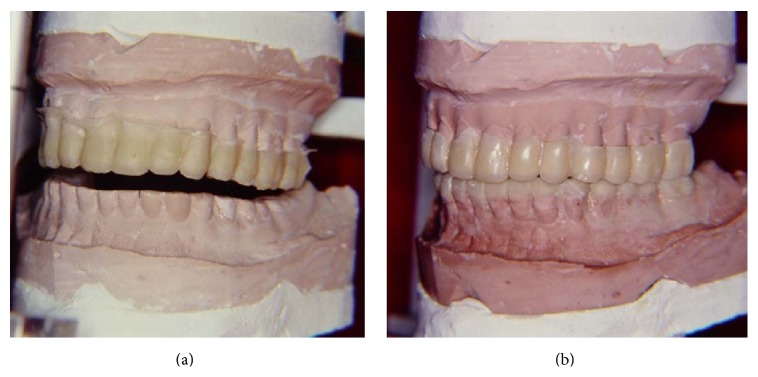
(a) Maxillary occlusal acrylic splint positioned in the second master cast set. (b) Waxing in the mandibular cast for manufacturing the second occlusal acrylic splint.

The first mandibular master cast was performed to determine an increase of 7 mm in the OVD. After waxing the maxillary cast (Figure 3), an autopolymerizing acrylic resin (Vipi Cor, Dental Vipi, Pirassununga, São Paulo, Brazil) was used to manufacture an occlusal acrylic splint with an increase in OVD of 7 mm; then, an occlusal adjustment in the semiadjustable articulator was made. Next, the maxillary occlusal acrylic splint was positioned together with the second mandibular master cast (OVD = 14 mm) and waxing in the mandibular cast was performed to manufacture a second occlusal acrylic splint (Figure 4).

**Figure 5:**
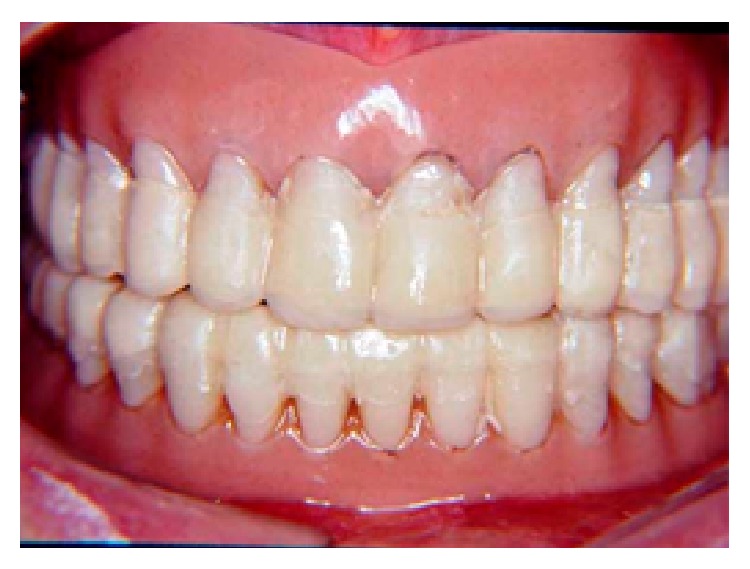
Installation of the mandibular occlusal splint after a 30-day period of adaptation.

**Figure 6:**
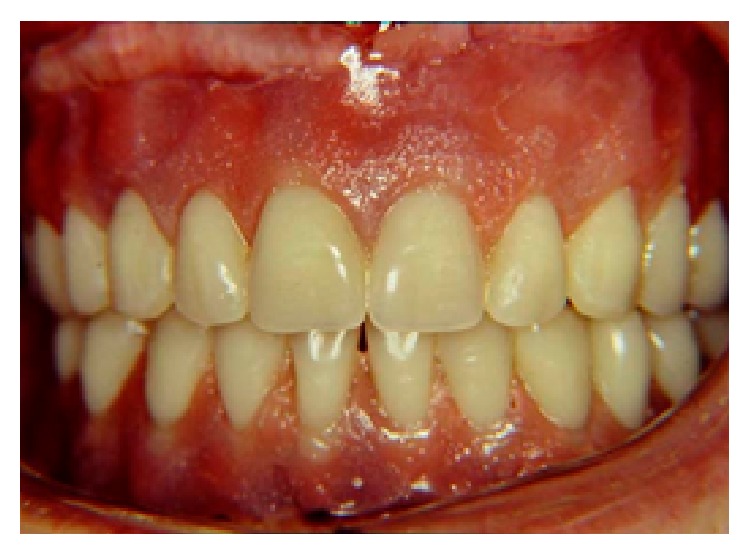
Installation of the new complete denture set after the adaptation of OVD of 14 mm.

The maxillary occlusal splint was first positioned on the old maxillary denture, fixed with composite resin, and an intraoral occlusal adjustment was made to evaluate the patient's ability to tolerate this increase of 7 mm for a period of 30 days. After this period of adaptation, the mandibular occlusal acrylic splint was fixed in the old mandibular denture (Figure 5), an intraoral occlusal adjustment was made, and a new period of 30 days was performed for the final adaptation of the patient to the appropriate OVD. Finally, after evaluating the adaptation of the patient to the increased OVD of 14 mm, all procedures for manufacturing a complete denture were performed and a new set was installed (Figure 6). This process allowed for the verification of the patient's ability to tolerate the proposed reestablishment of the OVD and evaluate her ability to adapt.

## 3. Discussion

The assessment and reestablishment of the OVD is considered an important factor in the treatment of complete dentures wearers [17]. The decrease in occlusal vertical dimension is a characteristic of complete denture wearers, mainly due to a marked resorption of the lower ridge with a resulting upward rotation of the mandible and an increase in mandibular prognathism [6]. Therefore, regular controls and early adjustments of the complete dentures are necessary in order to prevent marked changes in the jaw and occlusal relationships [6].

When controls and adjustments are not performed, changes of the OVD could interfere with the physiology of the masticatory system and the patient's ability to adapt [3, 18]. Matsuda et al. [4] conducted a study to identify how changes in the OVD can affect the sensory perception and activity of the brain in complete denture wearers. The present study [4] demonstrated that complete dentures with a lower vertical dimension decreased the masticatory force and a higher vertical dimension revealed an increase in psychological distress after gum chewing.

Although it has been observed that complete denture wearers can tolerate an increase in OVD with new dentures [19], the consequences of an inadequate OVD such as hyperactivity of the masticatory muscles and elevation in masticatory forces and TMDs have been related [3]. Therefore, it is prudent to evaluate the patient's OVD [1] and the reestablishing of the OVD must be based on the masticatory system's capacity to accept change and must be determined prior to fabrication of a new denture [1].

Most patients with old dentures and decrease in the OVD accept a gradual increase in the OVD, even if they are used to their old dentures [5]. This could be explained by the improvement in chewing functions and a gradual increase might be better tolerated than a reestablishment in one step [5]. It is desirable that the reestablishment of the OVD be carried out gradually, allowing the patient to adapt to their new OVD. The clinician should be capable of determining if a patient presents an inadequate OVD and then carry out an appropriate but reversible treatment before compromising the patient to a position that may not be able to function effectively [1, 13, 20].

The use of a diagnostic acrylic splint is a reversible therapy that could improve head and cervical spine postures and reestablish the OVD [21, 22], slowly and gradually allowing physiological postural repositioning, promoting relaxation of the suprahyoid muscles and retreatment of the hyoid bone [23], generating changes in craniocervical posture through the improvement of respiratory function [24], and improving the facial esthetic standard, resulting in a high self-esteem of edentulous patients.

In the present case report, a two-stage reestablishment of the OVD was performed due to the necessity to increase the OVD by 14 mm. This could allow for a reversible treatment if the patient does not adapt in the second increase phase. Therefore, a gradual reestablishment was necessary to evaluate the patient's ability to tolerate this increase, and it was demonstrated that this technique is an adequate alternative treatment.

## 4. Conclusion

The use of diagnostic occlusal acrylic splints on old complete dentures with an altered OVD is an effective and reversible treatment, gradually allowing reestablishment in the OVD, verifying the patient's ability to tolerate the proposed increase before manufacturing a new complete denture set.

## References

[1] Mays K. A. (2003). Reestablishing occlusal vertical dimension using a diagnostic treatment prosthesis in the edentulous patient: a clinical report. *Journal of Prosthodontics*.

[2] Yamashita S., Shimizu M., Katada H. (2015). A newly proposed method to predict optimum occlusal vertical dimension. *Journal of Prosthodontics*.

[3] Abduo J., Lyons K. (2012). Clinical considerations for increasing occlusal vertical dimension: a review. *Australian Dental Journal*.

[4] Matsuda R., Yoneyama Y., Morokuma M., Ohkubo C. (2014). The influence of vertical dimension of occlusion changes on the electroencephalograms of complete denture wearers. *Journal of Prosthodontic Research*.

[5] Fouda S. M., Al-Attar M. S., Virtanen J. I., Raustia A. (2014). Effect of patient's personality on satisfaction with their present complete denture and after increasing the occlusal vertical dimension: a study of edentulous egyptian patients. *International Journal of Dentistry*.

[6] Tallgren A., Lang B. R., Walker G. F., Ash M. M. (1980). Roentgen cephalometric analysis of ridge resorption and changes in jaw and occlusal relationships in immediate complete denture wearers. *Journal of Oral Rehabilitation*.

[7] Ormianer Z., Palty A. (2009). Altered vertical dimension of occlusion: a comparative retrospective pilot study of tooth and implant-supported restorations. *The International Journal of Oral & Maxillofacial Implants*.

[8] Gross M. D., Ormianer Z. (1994). A preliminary study on the effect of occlusal vertical dimension increase on mandibular postural rest position. *The International Journal of Prosthodontics*.

[9] Dantas E. M. (2012). The importance of restoring occlusal vertical dimension in the prosthetic rehabilitation. *Odonto*.

[10] Fujimoto M., Hayakawa I., Hirano S., Watanabe I. (2001). Changes in gait stability induced by alteration of mandibular position. *Journal of Medical and Dental Sciences*.

[11] Solow B., Tallgren A. (1976). Head posture and craniofacial morphology. *American Journal of Physical Anthropology*.

[12] Mohindra N. K., Bulman J. S. (2002). The effect of increasing vertical dimension of occlusion on facial aesthetics. *British Dental Journal*.

[13] Jahangiri L., Jang S. (2002). Onlay partial denture technique for assessment of adequate occlusal vertical dimension: a clinical report. *Journal of Prosthetic Dentistry*.

[14] Hansen C. A. (1985). Diagnostically restoring a reduced occlusal vertical dimension without permanently altering the existing dentures. *Journal of Prosthetic Dentistry*.

[15] de Souza R. F., Marra J., Pero A. C., Compagnoni M. A. (2007). Effect of denture fabrication and wear on closest speaking space and interocclusal distance during deglutition. *Journal of Prosthetic Dentistry*.

[16] Silverman M. M. (2001). The speaking method in measuring vertical dimension. *The Journal of Prosthetic Dentistry*.

[17] Rivera-Morales W. C., Mohl N. D. (1991). Relationship of occlusal vertical dimension to the health of the masticatory system. *The Journal of Prosthetic Dentistry*.

[18] Igić M., Krunić N., Aleksov L. (2015). Determination of vertical dimension of occlusion by using the phonetic vowel ‘O’ and ‘E’. *Vojnosanitetski Pregled*.

[19] Owen W. D., Douglas J. R. (1971). Near or full occlusal vertical dimension increase of severely reduced interarch distance in complete dentures. *The Journal of Prosthetic Dentistry*.

[20] Baba K., Tsukiyama Y., Clark G. T. (2000). Reliability, validity, and utility of various occlusal measurement methods and techniques. *The Journal of Prosthetic Dentistry*.

[21] Zanatta G., Silva W. A. B., Silva F. A., Ramos G. G., Casselli H. (2006). Assesment of painful symptomatology in patients with temporomandibular disorders by mean a combined experimental scale. *Brazilian Journal of Oral Sciences*.

[22] Casselli H., Landulpho A. B., Silva W. A. B., Silva F. A. (2007). Electrognathographic evaluations of rehabilitated edentulous patients. *Brazilian Oral Research*.

[23] Urbanowicz M. (1991). Alteration of vertical dimension and its effect on head and neck posture. *Cranio*.

[24] Vig P. S., Showfety K. J., Phillips C. (1980). Experimental manipulation of head posture. *American Journal of Orthodontics*.

